# Neonatal mortality from avoidable causes in the urban network: a spatial analysis, Brazil, 2020-2023

**DOI:** 10.1590/S2237-96222026v35e20250034.en

**Published:** 2026-05-14

**Authors:** Rivaldo Mauro Faria, Jean Ezequiel Limongi

**Affiliations:** ¹ Universidade Federal de Uberlândia, Instituto de Geografia, Uberlândia, MG, Brazil Universidade Federal de Uberlândia Instituto de Geografia Uberlândia MG Brazil

**Keywords:** Infant Mortality, Cause of Death, Infant, Newborn, Pregnancy, Spatial Analysis, Mortalidad Infantil, Causas de Muerte, Recién Nacido, Embarazo, Análisis Espacial

## Abstract

**Objective:**

To analyze spatial variations in the neonatal mortality rate due to avoidable causes within the Brazilian urban and regional network between 2020 and 2023.

**Methods:**

SA spatial analysis was conducted for Brazilian municipalities using data recorded in the Mortality Information System and the Live Birth Information System. The causes were defined according to the List of avoidable causes of death by interventions of the Brazilian National Health System, and mapped according to the classification of the urban network by the Brazilian Institute of Geography and Statistics. The neonatal mortality rate due to avoidable causes was estimated using the empirical Bayesian method. Analyses were performed using spatial autocorrelation techniques, through local and global Moran's indices.

**Results:**

A total of 65,183 neonatal deaths due to selected avoidable causes were recorded. Higher rates were found in the North (7.5 per 1,000 live births) and Northeast (7.0 per 1,000 live births) regions, while lower rates were observed in the South (4.9 per 1,000 live births) and Southeast (5.7 per 1,000 live births) regions. Rates were higher in local centers of the North (7.9 per 1,000 live births) and lower in the metropolises of the South (4.4 per 1,000 live births). Attention to women during pregnancy was the main avoidable cause (37.3%), followed by newborn attention (21.7%) and attention during childbirth (13.2%). The global Moran's index indicated spatial dependence of 0.240 (p-value 0.001).

**Conclusion:**

Spatial inequalities were observed in the neonatal mortality rate due to avoidable causes, with notably higher rates in local centers of the North and Northeast regions, indicating the need for preventive actions during pregnancy, childbirth, and newborn care.

## Resumen

**Objetivo:**

Analizar las variaciones espaciales de la tasa de mortalidad neonatal por causas evitables en la red urbana y regional brasileña entre 2020 y 2023.

**Métodos:**

Análisis espacial por municipios brasileños, realizado con datos registrados en el Sistema de Información sobre Mortalidad y en el Sistema de Información sobre Nacimientos Vivos. Se evaluaron las causas definidas en la Lista de causas de muertes evitables por intervenciones del Sistema Único de Salud, siendo mapeadas según la clasificación de la red urbana del Instituto Brasileño de Geografía y Estadística. La tasa de mortalidad neonatal por causas evitables se estimó mediante el método bayesiano empírico. Los análisis se realizaron con técnicas de autocorrelación espacial, a través de los índices de Moran local y global.

**Resultados:**

Se registraron 65.183 defunciones neonatales por causas evitables seleccionadas. Las tasas fueron mayores en las regiones Norte (7,5/1.000 nacimientos vivos) y Nordeste (7,0/1.000 nacimientos vivos) y menores en las regiones Sur (4,9/1.000 nacimientos vivos) y Sudeste (5,7/1.000 nacimientos vivos). Las tasas fueron mayores en los centros locales del Norte (7,9/1.000 nacimientos vivos) y menores en las metrópolis del Sur (4,4/1.000 nacimientos vivos). La atención a la mujer durante el embarazo fue la principal causa evitable (37,3%), seguida de la atención al recién nacido (21,7%) y la atención en el parto (13,2%). El índice global de Moran mostró dependencia espacial de 0,240 (valor de p 0,001).

**Conclusión:**

Se observaron desigualdades espaciales en la tasa de mortalidad neonatal por causas evitables, con énfasis en las elevadas tasas de los centros locales de las regiones Norte y Nordeste, indicando la necesidad de acciones de prevención durante el embarazo, el parto y la atención al recién nacido.

**Ethical aspects**

This research used public domain anonymized databases.

## Introduction

The reduction of neonatal mortality (up to 27 days of life) is one of the greatest challenges for child health policies worldwide and in Brazil^1^^,^^2^, and it is also a goal of the Sustainable Development Objectives for 2030^1^. Most of these deaths could be avoided through appropriate actions during pregnancy, childbirth, and the first days of the newborn's life^3^^,^^4^. 

Access to health services-particularly prenatal care and neonatal intensive care units-has been identified as a key determinant in protecting mothers and newborns^5^^,^^6^. Prematurity and low birth weight have been recognized as risk factors for neonatal mortality globally and in Brazil^7^, being more prevalent among pregnant people aged 40 years or older, with fewer than six prenatal consultations, and with low educational levels^8^.

Between 2000 and 2018, there was a reduction in the neonatal mortality rate in Brazil^9^. However, the 2020 rate (8.7 per 1,000 live births) remained high when compared to developed countries or even to some neighboring regional countries (Chile: 4.4 per 1,000 live births; Argentina: 4.6 per 1,000 live births; and Uruguay: 4.1 per 1,000 live births)^10^. In 2022, the neonatal mortality rate in Brazil remained high for avoidable causes, especially in the socially more vulnerable regions of the North and Northeast^2^.

Neonatal mortality is closely related to regional inequalities in Brazil^11^. Pregnancy and childbirth conditions are worse in remote rural municipalities and in border areas^12^^,^^13^. The geographical accessibility of municipalities has been identified as a determinant for saving babies at risk^13^ and for implementing preventive actions during and after pregnancy^14^. However, the effectiveness of health networks depends directly on the structure of the Brazilian urban network, which may be more or less integrated and more or less unequal^11^. There are differences in maternal and child health conditions according to levels of urban centrality, since these influence municipal economic and human development and access to health services.

The geographical position of municipalities within the Brazilian network may be a determinant of child health^13^^,^^14^. The Brazilian urban network is heterogeneous and unequal; the conditions for preventing neonatal deaths may also vary according to urban typology (metropolis, medium-sized cities, local centers, among others)^15^. Therefore, spatial analysis can be an important tool for identifying patterns of neonatal mortality within the Brazilian urban network, helping to identify priority situations according to city typology. 

This study aimed to analyze spatial variations in the neonatal mortality rate due to avoidable causes within the Brazilian urban and regional network between 2020 and 2023.

## Methods

Study design

This spatial analysis, conducted by Brazilian municipalities, is based on records of neonatal deaths due to avoidable causes during the 2020-2023 period. The analysis period was defined to include the most recent available data. Grouping the data into four years was a strategy to achieve greater rate stability for municipalities with small populations.

Study setting and unit of analysis

Brazilian municipalities were classified by regions (North, Northeast, Southeast, South, and Central-West) and by levels of centrality or urban hierarchy (metropolis, regional capital, sub-regional center, zone center, and local center). This classification was adopted by the Brazilian Institute of Geography and Statistics (*Instituto Brasileiro de Geografia e Estatística*, IBGE), based on the study of the regions of influence of Brazilian cities^16^. In that study, the Institute classified 4,899 urban centralities. Municipalities located in continuous urban areas with intense spatial interaction were grouped into 617 population arrangements. In such cases, the classification of urban hierarchy was applied to each population arrangement. For the population arrangements, the rate was calculated for the corresponding group of municipalities.

Data sources

Data were collected from the Mortality Information System (*Sistema de Informações sobre Mortalidade*, SIM) and the Live Birth Information System (*Sistema de Informações sobre Nascidos Vivos*, SINASC) on 10 September 2024. Deaths were evaluated according to the mother's municipality of residence. Deaths from avoidable causes were defined according to the List of avoidable causes of death by interventions of the Brazilian National Health System (*Sistema Único de Saúde*, SUS), updated in 2010^17^. 

These causes were grouped into four categories: i) avoidable through adequate care for women during pregnancy; ii) avoidable through adequate care for women during childbirth; iii) avoidable through adequate care for newborns; and iv) avoidable through health promotion actions, and appropriate diagnosis and treatment. Deaths avoidable by immunization actions, deaths from ill-defined causes, and other deaths not clearly avoidable were not included in the analysis. The latter two groups were excluded due to the imprecision in determining avoidability, and the first group was excluded due to the small number of cases.

Measurement

The neonatal mortality rate due to selected avoidable causes was estimated using the empirical Bayesian method^18^. This method has been recommended to reduce random fluctuations in rates in population-based studies involving a large number of areas and the possibility of a small number of events per area, as was the case in this study. The empirical Bayesian approach assumed that the rate *ϴ*_*i*_ was a random variable, containing a mean *μ*_*i*_ and a variance σ_1_^2^. To calculate it, the observed rate (*t*_*i*_ ), the mean (*μ*_*i*_ ), and the weight (*w*_*i*_ ) of the indicator for each municipality were combined, as described in the following equation.

$${ϴ}_{i}=w_{i}t_{i}+(1-w_{i})\;\mu_{i}$$

The factor *w*_*i*_ was defined as:

$$w_{i}=\;\frac{\sigma_{i}^{2}}{\sigma_{i}^{2}+u_{i}/\;n_{i}}$$

Through this method, the municipalities had their crude indicators re-estimated by applying a weighted mean, where the factor (*w*_*i*_ ) or confidence weight varied according to the size of the study population, being smaller for smaller populations and larger for larger ones. The same technique was applied to estimate the individual indicators related to avoidable causes.

The confidence intervals for the estimated rates were calculated using the Wilson score method, without continuity correction^19^. An online calculator was used for the confidence interval calculations^20^. This method provided more accurate results and prevented values from occurring outside the range of 0 to 1. In addition, it yielded safer and more conservative results, regardless of sample size or when the observed proportion was close to 0 or 1^19^. 

Statistical methods

The analyses were carried out using exploratory spatial data analysis techniques. Choropleth maps were created with five classes distributed according to the natural breaks method, as recommended by Ferreira^21^. To identify spatial patterns, global and local indicators of spatial autocorrelation were applied. For this purpose, a spatial weights matrix was constructed using the Queen contiguity criterion, in which neighboring units share either a common border or a vertex. 

In this study, first-order contiguity was used, considering only direct neighbors. This method was employed to include any form of physical contact and is considered appropriate for population-based studies^22^. Based on the defined form of spatial relationship (in this case, neighborhood-based), it was possible to measure global and local Moran's indices, as well as to perform the pseudo-significance (p-value) test. 

In this study, p-value randomization was performed with 999 permutations, the standard number of tests considered adequate for descriptive spatial analyses. The results were presented in a Moran scatterplot, showing quadrants of spatial relationships: Q1 (high-high) and Q2 (low-low), indicating positive spatial autocorrelation between the values of a variable and the mean of its neighbors; and Q3 (high-low) and Q4 (low-high), indicating negative spatial autocorrelation, meaning that the values of a variable in a given locality were not spatially related to those of neighboring areas^18^.

Descriptive statistical techniques were also used to calculate measures of central tendency (arithmetic mean) and variability or dispersion (standard deviation and coefficient of variation). Cartographic elaboration was performed using the ArcGIS software (Esri), accessed through the Federal University of Santa Maria. The digital cartographic base, in *shapefile* format, was obtained from the Brazilian Institute of Geography and Statistics (IBGE), at a 1:2,500,000 scale.

## Results

Between 2020 and 2023, 88,257 neonatal deaths were recorded in Brazil, of which 65,183 (73.9%) could have been avoided through adequate care for women during pregnancy (37.3%), adequate care during childbirth (13.2%), adequate care for the newborn (21.7%), and health promotion, diagnostic, and appropriate treatment actions (1.7%) (Table 1). 

The neonatal mortality rate due to selected avoidable causes was 6.2 per 1,000 live births, with a range from 3.4 to 13.6 per 1,000 live births (Figure 1A). Rates were higher in the North and Northeast regions and lower in the Southeast, South, and Central-West regions (Figure 1A). The global Moran's index indicated spatial dependence (0.240; p-value 0.001), with clusters of high rates (high-high) in the North and Northeast regions and clusters of low rates (low-low) in the South and Southeast, including areas of the Central-West (Figure 1B). 

**Table 1:** Relative distribution and 95% confidence interval (95%CI) of avoidable neonatal deaths, according to the interventions analyzed in the study, by region. Brazil, 2020-2023 (n=65,183)

Regions	Adequate care for women during pregnancy (95%CI)	Adequate attention to the woman in childbirth (95%CI)	Adequate care for the newborn (95%CI)	Appropriate preventive, diagnostic, and treatment actions (95%CI)
North	35.2 (34.3; 36.0)	14.0 (13.4; 14.7)	24.8 (24.0; 25.5)	2.2 (1.9; 2.5)
Northeast	35.6 (35.1; 36.2)	14.5 (14.1; 14.9)	23.2 (22.7; 23.7)	1.5 (1.3; 1.6)
Southeast	37.7 (37.2; 38.3)	13.2 (12.8; 13.6)	21.3 (20.8; 21.7)	1.4 (1.3; 1.6)
South	42.6 (41.6; 43.5)	10.2 (9.6; 10.8)	16.0 (15.3; 16.7)	1.7 (1.5; 2.0)
Central-West	38.5 (37.4; 39.6)	11.5 (10.8; 12.3)	20.5 (19.6; 21.4)	1.6 (1.4; 2.0)
Mean	37.3 (37.0; 37.7)	13.2 (13.0; 13.5)	21.7 (21.4; 21.9)	1.7 (1.5; 2.0)

**Figure 1:**
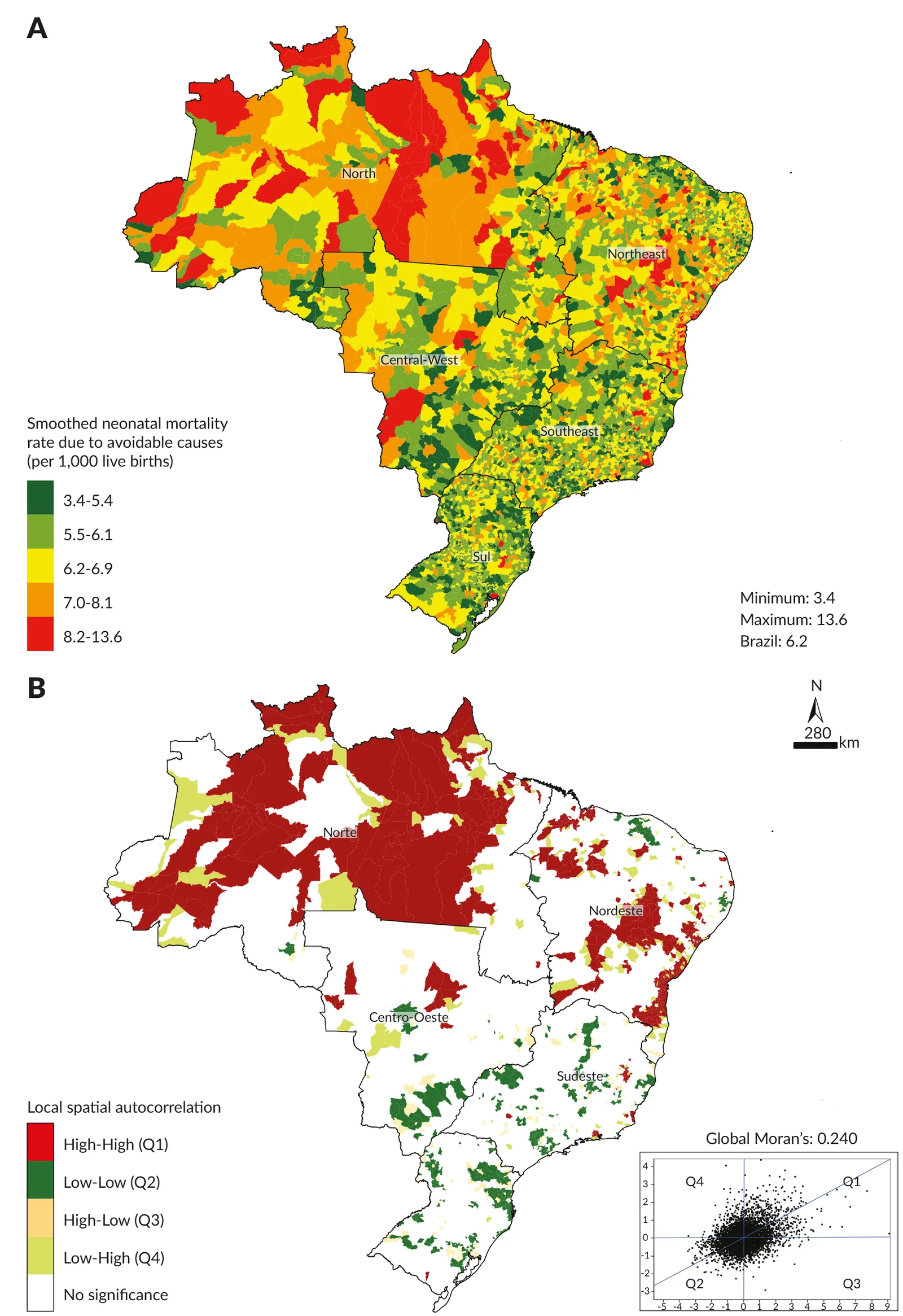
Smoothed neonatal mortality rate from avoidable causes (A) and local spatial autocorrelation of the neonatal mortality rate from avoidable causes (B), by municipality. Brazil, 2020-2023 (n=65,183)

The neonatal mortality rate due to selected avoidable causes was higher in the urban network of the North and Northeast (7.5 per 1,000 live births and 7.0 per 1,000 live births, respectively), particularly in local centers (Table 2). The lowest rates were found in the urban network of the Southeast and South regions (5.7 and 4.9 per 1,000 live births, respectively), notably in the metropolises of the South. The Central-West region presented an intermediate rate compared to the other regions (5.9 per 1,000 live births), with the lowest rates observed in the regional capitals.

The avoidable neonatal mortality rate related to adequate care for women during pregnancy was the highest among all causes evaluated. The mean ranged from 3.5 per 1,000 live births in the North to 2.9 per 1,000 live births in the Southeast and South regions (Table 3). The avoidable neonatal mortality rate related to adequate care for the newborn was the second highest. The mean ranged from 2.4 per 1,000 live births in the North to 1.1 per 1,000 live births in the South. The avoidable neonatal mortality rate due to adequate care during childbirth was highest in the North (mean 1.4 per 1,000 live births) and lowest in the South (mean 0.7 per 1,000 live births). The avoidable neonatal mortality rate, attributed to health promotion, diagnostic, and appropriate treatment actions, was highest in the North (mean 0.2 per 1,000 live births) and lowest in the other regions (mean 0.1 per 1,000 live births).

The coefficient of variation was higher in the inter-regional urban network than in the intra-regional urban network (Table 4). Within the intra-regional network, the greatest variation occurred in the neonatal mortality rate avoidable by health promotion, diagnostic, and appropriate treatment actions (56.5% in the North), followed by the neonatal mortality rate avoidable by adequate care for the newborn (22.7% in the Central-West). In the inter-regional urban network, the highest variations occurred among local centers (52.4% for the rate avoidable by preventive, diagnostic, and appropriate treatment actions) and metropolises (30.5% for the rate avoidable by adequate care for the newborn). The neonatal mortality rate avoidable by adequate care during childbirth also showed high variation among local centers and zone centers in the inter-regional urban network (28.4% and 26.8%, respectively).

## Discussion 

This study highlighted inequalities in the neonatal mortality rate due to selected avoidable causes within the Brazilian urban and regional network. Differences were evident, with higher rates in the North and Northeast regions, compared to lower rates in the South and Southeast. The Central-West presented an intermediate situation relative to the other regions. These results were consistent with previous spatial assessments conducted for the country as a whole^9^^,^^11^.

The neonatal mortality rate due to selected avoidable causes was higher in municipalities with a lower hierarchical level of the urban network in the North and Northeast regions. The same pattern was not observed in the more developed Southeast and South regions. Local centers in the North and Northeast were predominantly rural, characterized by poor transportation infrastructure and high poverty rates^23^. This hindered access to health services, particularly hospital childbirth and neonatal intensive care units. Difficulties in accessing physicians and health care services were observed in remote rural municipalities of the Northeast and the Amazon^24^. Being born in one's own municipality was considered a protective indicator for both the child and the mother in local centers^25^, as it allowed rapid access in urgent situations related to pregnancy and childbirth.

The main avoidable causes of neonatal mortality in Brazil were associated with pregnancy, childbirth care, and newborn care. Rates were higher in the Northeast and North and lower in the South and Southeast. This may be associated with difficult access to protective hospital services, whether for childbirth itself or in situations requiring specialized health equipment, such as neonatal intensive care units, which are essential for preterm and low birth weight newborns^5^. 

**Table 2:** Neonatal mortality rates from avoidable causes and 95% confidence intervals (95%CI), according to levels of urban centrality and region. Brazil, 2020-2023 (n=65,183)

Urban hierarchy	North (95%CI)	Northeast (95%CI)	Southeast (95%CI)	South (95%CI)	Central-West (95%CI)	Average (95%CI)
Metropolises	6.9 (6.5; 7.2)	6.8 (6.6; 7.0)	5.8 (5.7; 5.9)	4.4 (4.2; 4.6)	6.0 (5.8; 6.3)	5.9 (5.8; 6.0)
Regional capitals	7.6 (7.2; 7.9)	7.2 (7.0; 7.4)	5.8 (5.6; 6.0)	4.9 (4.7; 5.1)	5.1 (4.8; 5.5)	6.2 (6.1; 6.3)
Sub-regional centers	7.4 (7.0; 7.9)	6.6 (6.4; 6.9)	5.6 (5.4; 5.8)	4.9 (4.7; 5.2)	6.5 (6.1; 6.9)	6.0 (5.9; 6.1)
Zone centers	7.4 (6.8; 8.1)	7.1 (6.7; 7.4)	5.7 (5.4; 6.1)	5.3 (4.9; 5.7)	5.8 (5.3; 6.4)	6.3 (6.2; 6.5)
Local centers	7.9 (7.6; 8.1)	7.2 (7.1; 7.4)	5.7 (5.5; 5.9)	5.3 (5.0; 5.5)	6.1 (5.8; 6.5)	6.7 (6.6; 6.8)
Mean	7.5 (7.4; 7.7)	7.0 (7.0; 7.1)	5.7 (5.7; 5.8)	4.9 (4.8; 5.0)	5.9 (5.8; 6.1)	-

**Table 3:** Neonatal mortality rates from avoidable causes by interventions analyzed in the study, and 95% confidence intervals (95%CI), according to levels of urban centrality and region. Brazil, 2020-2023 (n=65,183)

Urban hierarchy	Adequate care for women during pregnancy (95%CI)	Adequate attention to the woman in childbirth (95%CI)	Adequate care for the newborn (95%CI)	Appropriate preventive, diagnostic, and treatment actions (95%CI)
North				
Metropolises	3.8 (3.5; 4.0)	0.1 (0.9; 1.1)	2.1 (1.9; 2.3)	0.1 (0.0; 0.1)
Regional capitals	3.9 (3.6; 4.1)	1.2 (1.0; 1.3)	2.4 (2.2; 2.6)	0.1 (0.1; 0.2)
Sub-regional centers	3.2 (2.9; 3.5)	1.3 (1.1; 1.4)	2.8 (2.6; 3.1)	0.1 (0.1; 0.2)
Zone centers	3.4 (2.9; 3.8)	1.4 (1.1; 1.7)	2.5 (2.2; 3.0)	0.2 (0.2; 0.3)
Local centers	3.2 (3.1; 3.4)	1.7 (1.6; 1.9)	2.5 (2.4; 2.7)	0.4 (0.3; 0.4)
Mean	3.5 (3.4; 3.6)	1.4 (1.3; 1.5)	2.4 (2.4; 2.5)	0.2 (0.2; 0.2)
Northeast				
Metropolises	3.8 (3.6; 4.0)	1.2 (1.1; 1.3)	1.7 (1.6; 1.8)	0.2 (0.1; 0.2)
Regional capitals	3.8 (3.7; 3.9)	1.2 (1.1; 1.3)	2.1 (1.9; 2.2)	0.1 (0.1; 0.1)
Sub-regional centers	2.9 (2.8; 3.1)	1.3 (1.2; 1.4)	2.3 (2.1; 2.4)	0.1 (0.1; 0.2)
Zone centers	3.0 (2.8; 3.2)	1.6 (1.4; 1.7)	2.3 (2.1; 2.5)	0.1 (0.1; 0.2)
Local centers	3.1 (3.0; 3.2)	1.5 (1.5; 1.6)	2.4 (2.4; 2.5)	0.1 (0.1; 0.2)
Mean	3.4 (3.3; 3.4)	1.4 (1.3; 1.4)	2.2 (2.1; 2.2)	0.1 (0.1; 0.2)
Southeast				
Metropolises	3.0 (2.9; 3.1)	1.1 (1.0; 1.1)	1.6 (1.5; 1.6)	0.1 (0.1; 0.1)
Regional capitals	3.2 (3.0; 3.3)	1.1 (1.0; 1.2)	1.5 (1.4; 1.6)	0.1 (0.1; 0.1)
Sub-regional centers	2.6 (2.5; 2.7)	0.9 (0.8; 0.9)	2.0 (1.9; 2.1)	0.1 (0.1; 0.2)
Zone centers	2.8 (2.5; 3.0)	1.1 (1.0; 1.3)	1.7 (1.5; 1.9)	0.1 (0.1; 0.2)
Local centers	2.8 (2.7; 3.0)	0.9 (0.8; 1.0)	1.8 (1.7; 2.0)	0.1 (0.1; 0.1)
Mean	2.9 (2.9; 3.0)	1.0 (1.0; 1.1)	1.7 (1.6; 1.7)	0.1 (0.1; 0.1)
South				
Metropolises	2.8 (2.7; 3.0)	0.7 (0.6; 0.8)	0.8 (0.7; 0.9)	0.1 (0.1; 0.1)
Regional capitals	3.1 (2.9; 3.3)	0.7 (0.6; 0.8)	1.0 (0.9; 1.1)	0.1 (0.1; 0.2)
Sub-regional centers	3.0 (2.8; 3.2)	0.6 (0.5; 0.7)	1.2 (1.1; 1.4)	0.1 (0.1; 0.2)
Zone centers	2.9 (2.6; 3.3)	0.7 (0.6; 0.9)	1.5 (1.2; 1.7)	0.2 (0.1; 0.3)
Local centers	2.9 (2.7; 3.1)	0.8 (0.7; 0.9)	1.4 (1.3; 1.6)	0.1 (0.1; 0.2)
Mean	2.9 (2.9; 3.0)	0.7 (0.7; 0.7)	1.1 (1.1; 1.2)	0.1 (0.1; 0.1)
Central-West				
Metropolises	3.9 (3.7; 4.1)	0.9 (0.8; 1.0)	1.1 (1.0; 1.2)	0.1 (0.1; 0.1)
Regional capitals	2.5 (2.3; 2.8)	0.7 (0.6; 0.9)	1.7 (1.5; 1.9)	0.2 (0.1; 0.2)
Sub-regional centers	3.1 (2.9; 3.4)	1.0 (0.8; 1.1)	2.3 (2.0; 2.5)	0.1 (0.1; 0.2)
Zone centers	2.5 (2.2; 2.9)	0.9 (0.7; 1.2)	2.2 (1.8; 2.5)	0.2 (0.1; 0.3)
Local centers	2.7 (2.5; 3.0)	1.2 (1.0; 1.4)	2.1 (1.9; 2.3)	0.2 (0.1; 0.2)
Mean	3.2 (3.1; 3.3)	0.9 (0.9; 1.0)	1.7 (1.6; 1.8)	0.1 (0.1; 0.2)

**Table 4:** Coefficient of variation of the avoidable neonatal mortality rate by interventions analyzed in the study, according to intra-regional and inter-regional urban networks. Brazil, 2020-2023 (n=65,183)

Regions	Adequate care for women during pregnancy	Adequate attention to the woman in childbirth	Adequate attention to the newborn	Appropriate preventive, diagnostic, and treatment actions
Intraregional urban network				
North	8.0	19.4	10.1	56.5
Northeast	11.5	12.6	12.3	14.2
Southeast	6.5	10.0	10.8	13.5
South	3.0	9.5	21.3	28.7
Central-West	17.2	15.1	22.7	22.4
Interregional urban network				
Metropolises	12.9	17.7	30.5	24.7
Regional capitals	15.0	21.6	27.8	21.1
Sub-regional centers	6.9	24.9	24.4	10.9
Zone centers	9.7	26.8	19.3	14.5
Local centers	6.4	28.4	19.8	52.4

The North also stood out for the high number of avoidable deaths related to health promotion, diagnostic, and appropriate treatment actions, much higher than in the other regions. This may result from lower coverage of health services, particularly primary health care services^24^. 

Care for women during pregnancy was the main cause of avoidable neonatal mortality in Brazil, with high rates across almost all regions, although even higher in the North, Northeast, and Central-West. Prenatal care is likely the most important type of care to prevent these deaths, allowing for the prevention of congenital syphilis, maternal conditions, pregnancy complications, fetal growth restriction, and malnutrition, among others. 

Inadequate prenatal care is directly and indirectly associated with most avoidable deaths related to pregnancy and childbirth^6^^,^^26^. Since 1990, Brazil has expanded access to prenatal care due to advances in primary health care policies. However, these results demonstrate the need to strengthen care policies throughout the entire pregnancy. Special attention is recommended for remote rural municipalities in less developed regions, where access to health services is very limited^25^^,^^27^. 

Avoidable neonatal mortality related to adequate care for the newborn was the second leading cause of neonatal mortality in Brazil. Most of these deaths occurred within the first six days of life (early neonatal period)^2^, and were strongly associated with disorders originating in the perinatal period^28^. Higher rates in the urban network of the North and Northeast indicate the need for protective measures during the perinatal period, whether related to primary care services or to medium- and high-complexity hospital services. This also involves consideration of the regional urban network, including the implementation of more effective transportation means in urgent and emergency situations, especially for riverine, Indigenous, *quilombola*, and other vulnerable populations. 

The results indicated the need to reduce neonatal mortality due to inadequate care for women during childbirth across the urban network of the North and Northeast regions, particularly in local centers. One policy adopted to address this challenge in Brazil was the Stork Network (*Rede Cegonha*)^29^, a government program created in 2011 to expand access to hospital childbirth and reduce maternal and infant mortality. However, although it had positive effects in reducing health inequalities in Brazil^30^, the Stork Network did not effectively reach remote rural municipalities. Expanding this access is essential to prevent intrauterine hypoxia and birth asphyxia, neonatal aspiration syndrome, fetuses and newborns affected by placenta previa and other forms of placental abruption, and hemorrhage, all of which are associated with birth complications and maternal and perinatal mortality. 

Greater variability in the neonatal mortality rate due to selected avoidable causes was observed between regions than within the intra-regional urban network. This indicates larger differences between more and less developed regions. In other words, in less developed regions, the neonatal mortality rate due to selected avoidable causes was high across almost the entire urban network (although always higher in local centers). In contrast, in more developed regions, the opposite pattern was observed. 

There was greater homogeneity in rates in the more developed regions (South and Southeast) due to a more integrated urban network. Conversely, in the North, Northeast, and Central-West, greater variability in rates may be related to a more fragmented urban network. These findings are consistent with previous assessments of the influence of the urban network on population health according to the geographical accessibility of municipalities^13^^,^^15^. 

The data presented in this study are important for the development of regional policies to reduce neonatal mortality in Brazil, but are subject to the limitations of population-based research. One limitation was inaccuracies in the registration of data in the Live Birth Information System (SINASC) and the Mortality Information System (SIM). Another limitation was related to the study scale, which may have masked inequalities within municipalities. A third limitation was associated with the pandemic period, which directly and indirectly affected maternal and child health, with repercussions for neonatal mortality rates.

In conclusion, Brazil exhibits profound spatial inequalities in neonatal mortality due to avoidable causes. These inequalities vary according to the regional urban network and are particularly severe in less developed regions. There is a clear need for policies to reduce neonatal mortality due to avoidable causes in local centers of the North and Northeast, where living conditions and access to services are more limited. 

## Data Availability

The database and analysis codes used in the research are available at: https://doi.org/10.48331/SCIELODATA.AEGHQH
