# Supplementary material for: Neonatal mortality from avoidable causes in the urban network: a spatial analysis, Brazil, 2020-2023
Source: Epidemiol Serv Saude. 2026 May 14;35:e20250034. doi: 10.1590/S2237-96222026v35e20250034.en (PMC13686428; doi:10.1590/S2237-96222026v35e20250034.en)
Supplement: Translation

# Translation: Mortalidade neonatal por causas evitáveis na rede urbana: análise espacial, Brasil, 2020-2023

**DOI:** 10.1590/S2237-96222026v35e20250034.pt

**Authors:** Rivaldo Mauro Faria, Jean Ezequiel Limongi

**Affiliations:** ¹ Universidade Federal de Uberlândia, Instituto de Geografia, Uberlândia, MG, Brasil Universidade Federal de Uberlândia Instituto de Geografia Uberlândia MG Brasil

**Keywords:** Mortalidade Neonatal, Causas de Morte, Recém-nascido, Gravidez, Análise Espacial

## Abstract

**Objetivo:**

Analisar as variações espaciais da taxa de mortalidade neonatal por causas evitáveis na rede urbana e regional brasileira entre 2020 e 2023.

**Métodos:**

Análise espacial, por municípios brasileiros, realizada com dados registrados no Sistema de Informações sobre Mortalidade e no Sistema de Informações sobre Nascidos Vivos. Avaliaram-se as causas definidas na *Lista de causas de mortes evitáveis por intervenções do Sistema Único de Saúde*, sendo mapeadas conforme a classificação da rede urbana pelo Instituto Brasileiro de Geografia e Estatística. A taxa de mortalidade neonatal por causas evitáveis foi estimada com método bayesiano empírico. As análises foram realizadas com técnicas de autocorrelação espacial, através dos índices de Moran local e global.

**Resultados:**

Registraram-se 65.183 óbitos neonatais por causas evitáveis selecionadas. Foram maiores as taxas nas regiões Norte (7,5/1 mil nascidos vivos) e Nordeste (7,0/1 mil nascidos vivos) e menores nas regiões Sul (4,9/1 mil nascidos vivos) e Sudeste (5,7/1 mil nascidos vivos). As taxas foram maiores nos centros locais do Norte (7,9/1 mil nascidos vivos) e menores nas metrópoles do Sul (4,4/1 mil nascidos vivos). A atenção à mulher na gestação foi a principal causa evitável (37,3%), seguida da atenção ao recém-nascido (21,7%) e atenção no parto (13,2%). O índice de Moran global mostrou dependência espacial 0,240 (p-valor 0,001).

**Conclusão:**

Observaram-se desigualdades espaciais na taxa de mortalidade neonatal por causas evitáveis, com destaque para as elevadas taxas dos centros locais das regiões Norte e Nordeste, indicando a necessidade de ações de prevenção na gestação e no parto e ao recém-nascido.

**Aspectos éticos**

Esta pesquisa utilizou bancos de dados de domínio público e anonimizados.

## Introdução 

A redução da mortalidade neonatal (até 27 dias de vida) é um dos maiores desafios das políticas de saúde infantil no mundo e no Brasil^1^^,^^2^ e uma das metas dos objetivos do desenvolvimento sustentável para 2030^1^. A maior parte desses óbitos poderia ser evitada com ações adequadas na gravidez, no parto e nos primeiros dias de vida do recém-nascido^3^^,^^4^. 

O acesso aos serviços de saúde, principalmente ao pré-natal e à unidade de terapia intensiva neonatal, foi avaliado como determinante na proteção da mãe e do recém-nascido^5^^,^^6^. A prematuridade e o baixo peso as nascer foram considerados fatores de risco para a mortalidade neonatal no mundo e no Brasil^7^, sendo mais prevalentes em gestantes com idade igual ou superior a 40 anos, com menos de seis consultas pré-natal e baixa escolaridade^8^.

Houve redução da taxa de mortalidade neonatal no Brasil entre 2000 e 2018^9^. Entretanto, a taxa em 2020 (8,7/1 mil nascidos vivos) ainda era elevada quando comparada aos países desenvolvidos ou mesmo a alguns dos vizinhos regionais (Chile: 4,4/1 mil nascidos vivos; Argentina: 4,6/1 mil nascidos vivos; e Uruguai: 4,1/1 mil nascidos vivos)^10^. Em 2022, a taxa de mortalidade neonatal no Brasil continuava elevada para causas que eram evitáveis, sobretudo nas regiões socialmente mais vulneráveis do Norte e do Nordeste^2^.

A mortalidade neonatal tem considerável relação com as desigualdades regionais brasileiras^11^. As condições da gestação e do parto são piores nos municípios rurais remotos e nos municípios de fronteira^12^^,^^13^. A acessibilidade geográfica dos municípios foi avaliada como determinante para o salvamento de bebês em situação de risco^13^ e para a adoção de ações de prevenção durante e após a gravidez^14^. No entanto, a fluidez nas redes de saúde depende diretamente da situação da rede urbana brasileira, podendo ser mais ou menos integrada, como também mais ou menos desigual^11^. Há diferenciações nas condições de saúde materna e infantil conforme os níveis de centralidade urbana, pois dela depende o desenvolvimento econômico e humano dos municípios e o acesso aos serviços de saúde.

A posição geográfica dos municípios na rede brasileira pode ser determinante da saúde infantil^13^^,^^14^. A rede urbana brasileira é heterogênea e desigual; as condições de evitabilidade do óbito neonatal podem também variar conforme a tipologia urbana (metrópole, cidades médias, centros locais, entre outros)^15^. Por isso, a análise espacial pode ser ferramenta importante para identificar padrões da mortalidade neonatal na rede urbana brasileira, podendo-se identificar situações prioritárias conforme a tipologia de cidades. 

Este estudo teve como objetivo analisar as variações espaciais da taxa de mortalidade neonatal por causas evitáveis na rede urbana e regional brasileira entre 2020 e 2023.

## Métodos 

Delineamento do estudo

Trata-se de análise espacial, por municípios brasileiros, realizada com os registros dos óbitos neonatais por causas evitáveis no período 2020-2023. O período de análise foi definido por envolver os dados mais recentes disponíveis. O agrupamento em quatro anos foi o recurso usado para encontrar maior estabilidade das taxas para municípios de pequeno porte populacional.

Local do estudo e unidade de análise

Os municípios brasileiros foram classificados por regiões (Norte, Nordeste, Sudeste, Sul e Centro-Oeste) e níveis de centralidade ou hierarquia urbana (metrópole, capital regional, centro sub-regional, centro de zona e centro local). Essa foi a classificação adotada pelo Instituto Brasileiro de Geografia e Estatística, a partir do estudo das regiões de influência das cidades brasileiras^16^. Nesse estudo, o instituto classificou 4.899 centralidades urbanas. Os municípios localizados em áreas urbanas contínuas e de intensa fluidez foram agregados como arranjos populacionais, somando 617, sendo a classificação da hierarquia urbana válida, neste caso, para cada arranjo populacional. Para os arranjos populacionais, calculou-se a taxa daquele agrupamento de municípios.

Fonte dos dados

Os dados foram coletados do Sistema de Informações sobre Mortalidade e do Sistema de Informações sobre Nascidos Vivos em 10 de setembro de 2024. Foram avaliados os óbitos com indicação do município de residência da mãe. As mortes por causas evitáveis foram definidas pela *Lista de causas de mortes evitáveis por intervenções do Sistema Único de Saúde*, atualizada em 2010^17^. 

Essas causas foram reunidas em quatro grupos: i) evitáveis por adequada atenção à mulher na gestação; ii) evitáveis por adequada atenção à mulher no parto; iii) evitáveis por adequada atenção ao recém-nascido; e iv) evitáveis com ações de promoção, diagnóstico e tratamento adequado. Não foram avaliados os óbitos evitáveis por ações de imunização, causas mal definidas e demais causas não claramente evitáveis. Esses dois últimos grupos foram excluídos pela imprecisão da evitabilidade do óbito, e o primeiro, pelo pequeno número de casos.

Mensuração

A taxa de mortalidade neonatal por causas evitáveis selecionadas foi estimada com uso do método bayesiano empírico^18^. Esse método foi recomendado na redução de flutuações aleatórias de taxas em estudos de base populacional envolvendo o grande número de áreas e a possibilidade de número reduzido de eventos por área, como foi o caso deste estudo. O bayesiano empírico partiu do pressuposto de que a taxa *ϴ*_*i*_ foi uma variável aleatória, que continha média *μ*_*i*_ e variância σ_1_^2^. Para calculá-la, realizou-se a combinação entre a taxa observada (*t*_*i*_ ), a média (*μ*_*i*_ ) e o peso de confiança (*w*_*i*_ ) do indicador para cada município, conforme descrito na equação a seguir.

$${ϴ}_{i}=w_{i}t_{i}+(1-w_{i})\;\mu_{i}$$

O fator *w*_*i*_ foi dado por:

$$w_{i}=\;\frac{\sigma_{i}^{2}}{\sigma_{i}^{2}+u_{i}/\;n_{i}}$$

Através desse método, os municípios tiveram seus indicadores brutos reestimados pela aplicação da média ponderada, cujo fator (*w*_*i*_ ) ou peso de confiança variou conforme o tamanho da população em estudo, sendo sempre menor quanto menor for a população e vice-versa. Essa mesma técnica foi aplicada para estimar os indicadores individuais relativos às causas evitáveis.

Os intervalos de confiança das taxas estimadas foram calculados através do método de escore de Wilson, sem correção de continuidade^19^. Uma calculadora virtual foi utilizada para os cálculos dos intervalos^20^. Esse método apresentou resultados mais precisos e evitou a ocorrência de valores fora do intervalo de 0 e 1. Além disso, apresentou resultados mais seguros e conservadores independentemente do tamanho das amostras ou quando a proporção observada esteve próxima de 0 ou 1^19^. 

Métodos estatísticos

As análises foram realizadas com técnicas exploratórias de dados espaciais. Os mapas coropléticos foram feitos com cinco classes distribuídas através do método de quebras naturais, conforme orientação de Ferreira^21^. Para identificar padrões espaciais, aplicou-se o indicador global e local de autocorrelação espacial. Para isso, foi elaborada uma matriz de pesos espaciais, com uso de contiguidade do "tipo rainha", em que os vizinhos compartilharam uma borda comum ou um vértice. 

Neste estudo, foi utilizada a contiguidade de primeira ordem, que considerou apenas vizinhos diretos. Esse método foi utilizado para incluir qualquer forma de contato físico, sendo adequado em estudos populacionais^22^. A partir da definição da forma de relacionamento espacial avaliado (neste caso, baseado na vizinhança), foi possível medir os índices de Moran global e local, assim como realizar o teste de pseudossignificância (valor-p). 

Neste estudo, a randonização do valor-p foi feita com 999 permutações (número de teste padrão considerado adequado para análises espaciais descritivas). O resultado foi expresso em um diagrama de espalhamento de Moran, com quadrantes de relacionamentos espaciais: Q1 (alto-alto) e Q2 (baixo-baixo), que indicaram autocorrelação espacial positiva entre os valores de uma variável e a média dos vizinhos; e Q3 (alto-baixo) e Q4 (baixo-alto), que indicaram autocorrelação espacial negativa, ou seja, os valores de uma variável em determinada localidade não se relacionaram espacialmente com os vizinhos^18^.

Foram usadas também técnicas de estatística descritiva para calcular medidas de tendência central (média aritmética) e variabilidade ou dispersão (desvio-padrão e coeficiente de variação). A elaboração cartográfica foi realizada no programa ArcGIS, da empresa Esri, de acesso pela Universidade Federal de Santa Maria. A base cartográfica digital, em formato *shapefile*, foi obtida no Instituto Brasileiro de Geografia e Estatística, em escala 1:2.500.000.

## Resultados 

Entre 2020 e 2023, foram registrados 88.257 óbitos no período neonatal no Brasil, dos quais 65.183 (73,9%) poderiam ter sido evitados por adequada atenção à mulher na gestação (37,3%), adequada atenção ao parto (13,2%), adequada atenção ao recém-nascido (21,7%) e por ações de promoção, diagnóstico e tratamento adequado (1,7%) (Tabela 1). 

A taxa de mortalidade neonatal por causas evitáveis selecionadas foi 6,2/1 mil nascidos vivos e variou entre 3,4/1 mil nascidos vivos e 13,6/1 mil nascidos vivos (Figura 1A). Foram mais elevadas as taxas no Norte e no Nordeste e menores as taxas nas regiões Sudeste, Sul e Centro-Oeste (Figura 1A). O índice de Moran global mostrou dependência espacial (0,240; p-valor 0,001), com formação de aglomerados de taxas elevadas (alto-alto) nas regiões Norte e Nordeste e aglomerados de baixas taxas (baixo-baixo) no Sul e no Sudeste, incluindo áreas do Centro-Oeste (Figura 1B). 

**Tabela 1:** Distribuição relativa e intervalo de confiança (IC95%) dos óbitos neonatais evitáveis, segundo as intervenções analisadas no estudo, por região. Brasil, 2020-2023 (n=65.183)

Regiões	Adequada atenção à mulher na gestação (IC95%)	Adequada atenção à mulher no parto (IC95%)	Adequada atenção ao recém-nascido (IC95%)	Ações adequadas de prevenção, diagnóstico e tratamento adequado (IC95%)
Norte	35,2 (34,3; 36,0)	14,0 (13,4; 14,7)	24,8 (24,0; 25,5)	2,2 (1,9; 2,5)
Nordeste	35,6 (35,1; 36,2)	14,5 (14,1; 14,9)	23,2 (22,7; 23,7)	1,5 (1,3; 1,6)
Sudeste	37,7 (37,2; 38,3)	13,2 (12,8; 13,6)	21,3 (20,8; 21,7)	1,4 (1,3; 1,6)
Sul	42,6 (41,6; 43,5)	10,2 (9,6; 10,8)	16,0 (15,3; 16,7)	1,7 (1,5; 2,0)
Centro-Oeste	38,5 (37,4; 39,6)	11,5 (10,8; 12,3)	20,5 (19,6; 21,4)	1,6 (1,4; 2,0)
Média	37,3 (37,0; 37,7)	13,2 (13,0; 13,5)	21,7 (21,4; 21,9)	1,7 (1,5; 2,0)

**Figura 1:** Taxa suavizada de mortalidade neonatal por causas evitáveis (A) e autocorrelação espacial local da taxa de mortalidade neonatal por causas evitáveis (B) por município. Brasil, 2020-2023 (n=65.183)

A taxa de mortalidade neonatal por causas evitáveis selecionadas foi mais elevada na rede urbana do Norte e do Nordeste (7,5/1 mil nascidos vivos e 7,0/1 mil nascidos vivos), sobretudo nos centros locais (Tabela 2). As menores taxas foram encontradas na rede urbana das regiões Sudeste e Sul (5,7/1 mil nascidos vivos e 4,9/1 mil nascidos vivos), com destaque para as metrópoles da região Sul. O Centro-Oeste apresentou taxa intermediária em relação às demais regiões (5,9/1 mil nascidos vivos), sendo suas taxas mais baixas nas capitais regionais.

A taxa de mortalidade evitável por adequada atenção à mulher na gestação foi maior entre todas as causas avaliadas. A média variou entre 3,5/1 mil nascidos vivos no Norte e 2,9/1 mil nascidos vivos nas regiões Sudeste e Sul (Tabela 3). A taxa de mortalidade neonatal evitável por adequada atenção ao recém-nascido foi a segunda mais elevada. A média variou entre 2,4/1 mil nascidos vivos no Norte e 1,1/1 mil nascidos vivos no Sul. A taxa de mortalidade neonatal evitável por adequada atenção à mulher no parto foi maior no Norte (média de 1,4/1 mil nascidos vivos) e menor no Sul (média de 0,7/1 mil nascidos vivos). A taxa de mortalidade neonatal evitável por ações de prevenção, diagnóstico e tratamento adequado foi maior no Norte (média de 0,2/1 mil nascidos vivos), e menor nas demais regiões (média de 0,1/1 mil nascidos vivos).

O coeficiente de variação foi maior na rede urbana inter-regional do que na rede urbana intrarregional (Tabela 4). Na rede intrarregional, as maiores variações ocorreram na taxa de mortalidade neonatal evitável por ações de prevenção, diagnóstico e tratamento adequado (56,5% no Norte), seguida da taxa de mortalidade neonatal evitável por adequada atenção ao recém-nascido (22,7% no Centro-Oeste). Na rede urbana inter-regional, as maiores variações ocorreram entre centros locais (52,4% para a taxa de mortalidade neonatal evitável por ações de prevenção, diagnóstico e tratamento adequado) e metrópoles (30,5% para a da taxa de mortalidade neonatal evitável por adequada atenção ao recém-nascido). A taxa de mortalidade neonatal evitável por adequada atenção ao parto apresentou variação também elevada entre centros locais e centros de zona na rede urbana inter-regional (28,4% e 26,8%).

## Discussão 

Este estudo apontou as desigualdades na taxa de mortalidade neonatal por causas evitáveis selecionadas na rede urbana e regional brasileira. As diferenças foram notadas pelas elevadas taxas nas regiões Norte e Nordeste, comparativamente às taxas mais baixas no Sul e no Sudeste. O Centro-Oeste apresentou situação intermediária em relação às demais. Esses resultados foram condizentes com avaliações espaciais já feitas para o conjunto do país^9^^,^^11^.

A taxa de mortalidade neonatal por causas evitáveis selecionadas foi maior quanto menor foi o nível hierárquico da rede urbana das regiões Norte e Nordeste. O mesmo comportamento não ocorreu com as regiões mais desenvolvidas do Sudeste e do Sul. Os centros locais das regiões Norte e Nordeste foram predominantemente rurais, marcados pela precária infraestrutura de transporte e elevados índices de pobreza^23^. Isso dificultou o acesso aos serviços de saúde, principalmente ao parto hospitalar e às unidades de terapia intensiva neonatal. Observou-se dificuldade de acesso ao médico e aos serviços de atenção à saúde em municípios rurais remotos do Nordeste e da Amazônia^24^. Nascer no próprio município foi avaliado como indicador de proteção da criança e da mãe em centros locais^25^, pois permitiu o acesso rápido em situações emergenciais associadas à gestação e ao parto.

As principais causas evitáveis da mortalidade neonatal no Brasil foram associadas à gestação, ao atendimento ao parto e ao recém-nascido. As taxas foram igualmente maiores nas regiões Nordeste e Norte e menores no Sul e no Sudeste. Isso pôde estar associado à dificuldade de acesso aos serviços hospitalares de proteção, seja para a realização do próprio parto, seja em situações em que se exigem equipamentos de saúde, como unidades de terapia intensiva neonatal, reconhecida como fundamental para nascidos prematuros e com baixo peso^5^. 

**Tabela 2:** Taxas e intervalos de confiança de 95% (IC95%) da mortalidade neonatal por causas evitáveis, segundo níveis de centralidade urbana e região. Brasil, 2020-2023 (n=65.183)

Hierarquia urbana	Norte (IC95%)	Nordeste (IC95%)	Sudeste (IC95%)	Sul (IC95%)	Centro-Oeste (IC95%)	Média (IC95%)
Metrópoles	6,9 (6,5; 7,2)	6,8 (6,6; 7,0)	5,8 (5,7; 5,9)	4,4 (4,2; 4,6)	6,0 (5,8; 6,3)	5,9 (5,8; 6,0)
Capitais regionais	7,6 (7,2; 7,9)	7,2 (7,0; 7,4)	5,8 (5,6; 6,0)	4,9 (4,7; 5,1)	5,1 (4,8; 5,5)	6,2 (6,1; 6,3)
Centros sub-regionais	7,4 (7,0; 7,9)	6,6 (6,4; 6,9)	5,6 (5,4; 5,8)	4,9 (4,7; 5,2)	6,5 (6,1; 6,9)	6,0 (5,9; 6,1)
Centros de zona	7,4 (6,8; 8,1)	7,1 (6,7; 7,4)	5,7 (5,4; 6,1)	5,3 (4,9; 5,7)	5,8 (5,3; 6,4)	6,3 (6,2; 6,5)
Centros locais	7,9 (7,6; 8,1)	7,2 (7,1; 7,4)	5,7 (5,5; 5,9)	5,3 (5,0; 5,5)	6,1 (5,8; 6,5)	6,7 (6,6; 6,8)
Média	7,5 (7,4; 7,7)	7,0 (7,0; 7,1)	5,7 (5,7; 5,8)	4,9 (4,8; 5,0)	5,9 (5,8; 6,1)	-

**Tabela 3:** Taxas e intervalos de confiança de 95% (IC95%) da mortalidade neonatal por causas evitáveis pelas intervenções analisadas no estudo, segundo nível de centralidade urbana e região. Brasil, 2020-2023 (n=65.183)

Hierarquia urbana	Adequada atenção à mulher na gestação (IC95%)	Adequada atenção à mulher no parto (IC95%)	Adequada atenção ao recém-nascido (IC95%)	Ações adequadas de prevenção, diagnóstico e tratamento adequado (IC95%)
Norte				
Metrópoles	3,8 (3,5; 4,0)	0,1 (0,9; 1,1)	2,1 (1,9; 2,3)	0,1 (0,0; 0,1)
Capitais regionais	3,9 (3,6; 4,1)	1,2 (1,0; 1,3)	2,4 (2,2; 2,6)	0,1 (0,1; 0,2)
Centros sub-regionais	3,2 (2,9; 3,5)	1,3 (1,1; 1,4)	2,8 (2,6; 3,1)	0,1 (0,1; 0,2)
Centros de zona	3,4 (2,9; 3,8)	1,4 (1,1; 1,7)	2,5 (2,2; 3,0)	0,2 (0,2; 0,3)
Centros locais	3,2 (3,1; 3,4)	1,7 (1,6; 1,9)	2,5 (2,4; 2,7)	0,4 (0,3; 0,4)
Média	3,5 (3,4; 3,6)	1,4 (1,3; 1,5)	2,4 (2,4; 2,5)	0,2 (0,2; 0,2)
Nordeste				
Metrópoles	3,8 (3,6; 4,0)	1,2 (1,1; 1,3)	1,7 (1,6; 1,8)	0,2 (0,1; 0,2)
Capitais regionais	3,8 (3,7; 3,9)	1,2 (1,1; 1,3)	2,1 (1,9; 2,2)	0,1 (0,1; 0,1)
Centros sub-regionais	2,9 (2,8; 3,1)	1,3 (1,2; 1,4)	2,3 (2,1; 2,4)	0,1 (0,1; 0,2)
Centros de zona	3,0 (2,8; 3,2)	1,6 (1,4; 1,7)	2,3 (2,1; 2,5)	0,1 (0,1; 0,2)
Centros locais	3,1 (3,0; 3,2)	1,5 (1,5; 1,6)	2,4 (2,4; 2,5)	0,1 (0,1; 0,2)
Média	3,4 (3,3; 3,4)	1,4 (1,3; 1,4)	2,2 (2,1; 2,2)	0,1 (0,1; 0,2)
Sudeste				
Metrópoles	3,0 (2,9; 3,1)	1,1 (1,0; 1,1)	1,6 (1,5; 1,6)	0,1 (0,1; 0,1)
Capitais regionais	3,2 (3,0; 3,3)	1,1 (1,0; 1,2)	1,5 (1,4; 1,6)	0,1 (0,1; 0,1)
Centros sub-regionais	2,6 (2,5; 2,7)	0,9 (0,8; 0,9)	2,0 (1,9; 2,1)	0,1 (0,1; 0,2)
Centros de zona	2,8 (2,5; 3,0)	1,1 (1,0; 1,3)	1,7 (1,5; 1,9)	0,1 (0,1; 0,2)
Centros locais	2,8 (2,7; 3,0)	0,9 (0,8; 1,0)	1,8 (1,7; 2,0)	0,1 (0,1; 0,1)
Média	2,9 (2,9; 3,0)	1,0 (1,0; 1,1)	1,7 (1,6; 1,7)	0,1 (0,1; 0,1)
Sul				
Metrópoles	2,8 (2,7; 3,0)	0,7 (0,6; 0,8)	0,8 (0,7; 0,9)	0,1 (0,1; 0,1)
Capitais regionais	3,1 (2,9; 3,3)	0,7 (0,6; 0,8)	1,0 (0,9; 1,1)	0,1 (0,1; 0,2)
Centros sub-regionais	3,0 (2,8; 3,2)	0,6 (0,5; 0,7)	1,2 (1,1; 1,4)	0,1 (0,1; 0,2)
Centros de zona	2,9 (2,6; 3,3)	0,7 (0,6; 0,9)	1,5 (1,2; 1,7)	0,2 (0,1; 0,3)
Centros locais	2,9 (2,7; 3,1)	0,8 (0,7; 0,9)	1,4 (1,3; 1,6)	0,1 (0,1; 0,2)
Média	2,9 (2,9; 3,0)	0,7 (0,7; 0,7)	1,1 (1,1; 1,2)	0,1 (0,1; 0,1)
Centro-Oeste				
Metrópoles	3,9 (3,7; 4,1)	0,9 (0,8; 1,0)	1,1 (1,0; 1,2)	0,1 (0,1; 0,1)
Capitais regionais	2,5 (2,3; 2,8)	0,7 (0,6; 0,9)	1,7 (1,5; 1,9)	0,2 (0,1; 0,2)
Centros sub-regionais	3,1 (2,9; 3,4)	1,0 (0,8; 1,1)	2,3 (2.0; 2,5)	0,1 (0,1; 0,2)
Centros de zona	2,5 (2,2; 2,9)	0,9 (0,7; 1,2)	2,2 (1,8; 2,5)	0,2 (0,1; 0,3)
Centros locais	2,7 (2,5; 3,0)	1,2 (1,0; 1,4)	2,1 (1,9; 2,3)	0,2 (0,1; 0,2)
Média	3,2 (3,1; 3,3)	0,9 (0,9; 1,0)	1,7 (1,6; 1,8)	0,1 (0,1; 0,2)

**Tabela 4:** Coeficiente de variação da taxa de mortalidade neonatal evitável pelas intervenções analisadas no estudo, segundo a rede urbana intrarregional e inter-regional. Brasil, 2020-2023 (n=65.183)

Regiões	Adequada atenção à mulher na gestação	Adequada atenção à mulher no parto	Adequada atenção ao recém-nascido	Ações adequadas de prevenção, diagnóstico e tratamento adequado
Rede urbana intrarregional				
Norte	8,0	19,4	10,1	56,5
Nordeste	11,5	12,6	12,3	14,2
Sudeste	6,5	10,0	10,8	13,5
Sul	3,0	9,5	21,3	28,7
Centro-Oeste	17,2	15,1	22,7	22,4
Rede urbana inter-regional				
Metrópoles	12,9	17,7	30,5	24,7
Capitais regionais	15,0	21,6	27,8	21,1
Centros sub-regionais	6,9	24,9	24,4	10,9
Centros de zona	9,7	26,8	19,3	14,5
Centros locais	6,4	28,4	19,8	52,4

O Norte se destacou também pelo elevado número de óbitos evitáveis associados às ações de proteção, diagnóstico e tratamento adequado, muito maior que as demais regiões do país. Isso pôde ser resultado de menor cobertura dos serviços de saúde, sobretudo de serviços de atenção primária à saúde^24^. 

A atenção à mulher na gestação foi a principal causa de mortalidade neonatal evitável no Brasil, sendo a taxa elevada em quase todas as regiões brasileiras, embora ainda mais elevada nas regiões Norte, Nordeste e Centro-Oeste. O pré-natal talvez seja o tipo de atenção mais importante para evitar esse óbito, permitindo prevenir sífilis congênita, afecções maternas, complicações da gravidez, crescimento fetal retardado e desnutrição, entre outros. 

O pré-natal inadequado está associado direta e indiretamente à maior parte dos óbitos evitáveis ligados à gestação e ao parto^6^^,^^26^. A partir de 1990, o Brasil ampliou o acesso ao pré-natal, graças ao avanço das políticas de atenção primária. Contudo, esses resultados mostraram a necessidade de ampliação das políticas de atenção durante todo o período gestacional. De maneira especial, recomenda-se atenção aos municípios rurais remotos das regiões menos desenvolvidas, onde esse acesso é muito precário^25^^,^^27^. 

A mortalidade evitável por adequada atenção ao recém-nascido foi a segunda maior causa de mortalidade neonatal no Brasil. A maior parte desses óbitos ocorreu até os primeiros seis dias de vida (neonatal precoce)^2^, sendo muito relacionados com transtornos originados no período perinatal^28^. As taxas mais elevadas na rede urbana do Norte e do Nordeste indicaram a necessidade de adoção de medidas protetivas no período perinatal, sejam elas relacionadas aos serviços de atenção primária, sejam elas relacionadas aos serviços hospitalares de média e alta complexidade. Isso passa também pela consideração da rede urbana regional, com a implementação de meios de transporte mais eficazes em situações de urgência e emergência, especialmente para populações ribeirinhas, indígenas, quilombolas, entre outros grupos sociais vulneráveis. 

Os resultados indicaram a necessidade de redução da taxa de mortalidade neonatal evitável por adequada atenção à mulher no parto em toda a rede urbana das regiões Norte e Nordeste, em especial nos centros locais. Uma das políticas adotadas para enfrentar esse desafio no Brasil foi a Rede Cegonha^29^, programa governamental criado em 2011 para ampliar o acesso ao parto hospitalar e reduzir a mortalidade materna e infantil. Entretanto, embora tenha tido efeitos positivos na redução das desigualdades em saúde no Brasil^30^, a Rede Cegonha não logrou alcançar eficientemente os municípios rurais remotos do país. Ampliar esse acesso é fundamental para evitar hipóxia intrauterina e asfixia ao nascer, síndrome de aspiração neonatal, feto e recém-nascido afetados por placenta prévia e por outras formas de descolamento da placenta e hemorragia, que são associados a complicações no parto e mortalidade materna e perinatal. 

Observou-se maior variabilidade da taxa de mortalidade neonatal por causas evitáveis selecionadas entre as regiões do que na rede urbana intrarregional. Isso foi um indicativo das diferenças maiores entre as regiões mais e menos desenvolvidas. Em outros termos, nas regiões menos desenvolvidas, a taxa de mortalidade neonatal por causas evitáveis selecionadas foi elevada em quase toda sua rede urbana (embora sempre maior nos centros locais), como também o contrário ocorreu nas regiões mais desenvolvidas. 

Houve maior homogeneidade da taxa nas regiões mais desenvolvidas (Sul e Sudeste), em função da sua rede urbana mais integrada. Ao contrário, no Norte, no Nordeste e no Centro-Oeste, a maior variabilidade da taxa pôde estar relacionada com sua rede urbana mais fragmentada. Esses resultados foram condizentes com as avaliações sobre a influência da rede urbana nas condições de saúde da população em função da acessibilidade geográfica dos municípios^13^^,^^15^. 

Os dados apresentados neste trabalho são importantes para o desenvolvimento de políticas regionais de redução da mortalidade neonatal no Brasil e estiveram sujeitos às limitações de pesquisas de base populacional. Uma das limitações foram as imprecisões no registro desses dados no Sistema de Informações sobre Nascidos Vivos e no Sistema de Informações sobre Mortalidade. Outra limitação esteve relacionada com a escala do estudo, que pôde mascarar desigualdades internas aos municípios. Uma terceira limitação teve relação com o período pandêmico, o qual refletiu direta e indiretamente na saúde materna e infantil, com repercussões nas taxas de mortalidade neonatal.

Concluiu-se que o Brasil possui profundas desigualdades espaciais na mortalidade neonatal por causas evitáveis. Essas desigualdades são variáveis conforme a rede urbana regional, sendo particularmente mais graves nas regiões menos desenvolvidas. Destaca-se a necessidade de políticas de redução da mortalidade neonatal por causas evitáveis nos centros locais do Norte e do Nordeste, onde as condições de vida e de acesso aos serviços são mais precárias. 

## Data Availability

O banco de dados os códigos de análises utilizados na pesquisa estão disponíveis em: https://doi.org/10.48331/SCIELODATA.AEGHQH
